# Modulation of oxidative metabolism and quality preservation of strawberries by a CMC–alginate bilayer edible coating during cold storage

**DOI:** 10.1002/jsfa.71048

**Published:** 2026-09-03

**Authors:** Ilenia Pellicano, Riccardo Riggi, Elvira Ferrara, Danilo Cice, Margherita Modesti, Milena Petriccione, Andrea Bellincontro

**Affiliations:** ^1^ Department for Innovation of Biological, Agrofood and Forest Systems (DIBAF) University of Tuscia Viterbo Italy; ^2^ CREA‐Research Centre for Olive Fruit and Citrus Crops (CREA‐OFA) Caserta Italy

**Keywords:** antioxidant enzymes, ASCA, bioactive compounds, oxidative stress, postharvest quality

## Abstract

**BACKGROUND:**

Strawberries are highly perishable fruits characterized by rapid postharvest deterioration, leading to significant quality losses and reduced marketability. In this study, a bilayer edible coating based on carboxymethylcellulose and sodium alginate was evaluated for its ability to preserve strawberry quality during cold storage.

**METHODS:**

An integrated analytical approach combining physicochemical, colorimetric, biochemical, and enzymatic determinations with multivariate statistical analyses was adopted to investigate the physiological responses associated with fruit senescence.

**RESULTS:**

Compared to control fruit, coated strawberries exhibited reduced weight loss and higher firmness retention indicating a delay in ripening processes. The coating also increased the accumulation of bioactive compounds, leading to increased antioxidant activity. From a physiological perspective, coated fruit showed enhanced antioxidant enzyme activities and reduced oxidative damage, as confirmed by lower polyphenol oxidase, lipoxygenase, and malondialdehyde levels, suggesting improved membrane integrity and delayed senescence. ANOVA–simultaneous component analysis identified storage time as the major source of multivariate variation (61.99%), while treatment and the treatment × storage time interaction exerted significant, although less pronounced, effects on the physiological evolution of the fruit. Hierarchical clustering of the correlation matrix further highlighted coordinated relationships among antioxidant enzymes, oxidative damage markers, bioactive compounds, and quality‐related traits, providing an integrated view of the mechanisms underlying strawberry postharvest deterioration. Overall, the carboxymethylcellulose–alginate edible coating effectively delayed senescence by modulating oxidative metabolism and preserving fruit quality during refrigerated storage. The combined application of conventional analytical measurements and multivariate statistical approaches provides a comprehensive framework for understanding the physiological mechanisms underlying edible coating performance. © 2026 The Author(s). *Journal of the Science of Food and Agriculture* published by John Wiley & Sons Ltd on behalf of Society of Chemical Industry.

## INTRODUCTION

The strawberry (*Fragaria × ananassa*) is a false fruit belonging to the Rosaceae family, Rosoidea subfamily, *Fragaria* genus.^1^ Globally it is widely consumed due to its characteristic red color, juicy texture, sweet, fruity flavor, and nutritional quality.^2^, ^3^, ^4^

Strawberries represent an important source of micronutrients and antioxidants, such as minerals, vitamin C, folic acid^2^, ^5^, and phenolic compounds, including ellagic acid, anthocyanins, catechins, quercetin, and kaempferol.^2^, ^3^ Furthermore, their distinctive aroma is influenced by over 360 volatile organic compounds, which collectively define the aromatic profile of strawberry fruits.^6^, ^7^, ^8^, ^9^

Unfortunately, fresh strawberries have a short shelf life (approximately 5 days, when stored at 0–4 °C) due to their high respiration rate and low resistance to mechanical damage, as well as the lack of a skin to act as an external barrier to prevent water loss.^10^ This results in a rapid reduction in fruit quality and marketability.^11^ Moreover, they are particularly susceptible to microbial spoilage, such as by *Penicillium* spp.,^12^ posing a health hazard due to the possible production of mycotoxins.^13^

For these reasons, intervening through proper postharvest management can contribute to maintaining optimal organoleptic and commercial characteristics.^14^ Over the years, various methods have been studied to extend the shelf life of strawberries. These include low‐temperature storage,^15^ modified atmosphere packaging,^16^ irradiation using gamma ultraviolet rays^17^ or X‐rays.^18^

Among sustainable postharvest technologies, edible coatings have become one of the most widely investigated approaches for preserving the quality and extending the shelf life of fresh horticultural products. Edible coatings are made from natural biopolymers such as proteins, polysaccharides, and lipids, and they form a thin, edible layer on food surfaces.^19^, ^20^

These coatings represent a valid alternative to traditional preservation methods or to synthetic packaging, and they can extend shelf life and improve product quality and environmental sustainability. They provide several advantages, such as acting as a reinforcement layer on the external surface to prevent the loss of moisture, aromas, and ingredients from and between foods, thereby making a fruit's appearance more commercially appealing. They also facilitate the controlled exchange of essential gases involved in food respiration (carbon dioxide, oxygen, and ethylene),^19^, ^21^, ^22^ limit enzymatic browning, and enhance mechanical properties.^23^ In addition, coatings can improve the organoleptic characteristics of packaged foods by incorporating aromas and colorants, while also enabling the customization of surface properties (e.g. hydrophobicity, hydrophilicity). Coatings can also be enriched with antioxidant and antimicrobial compounds to limit oxidation and microbial proliferation. Different classes of biopolymers, including proteins, lipids, and polysaccharides, have been employed for this purpose, leading to the development of coatings suitable for strawberry preservation. Simple coatings, based on a single biopolymer, have shown some effectiveness in prolonging strawberry shelf life, but composite coatings, obtained by combining two or more biopolymers, are more widely used due to their superior physical properties. Common polymeric matrices include chitosan, carboxymethylcellulose (CMC), starches, gelatin, gums, and pectins, appreciated for their biodegradability, safety, and excellent coating performance.^24^, ^25^, ^26^, ^27^ Nanotechnology has further enhanced coating performance: the incorporation of cellulose nanocrystals, carnauba wax nanoemulsions,^28^ and bacterial cellulose nanofibers^29^ into biopolymer‐based matrices has significantly improved the physical and chemical stability of coatings, helping to preserve strawberry quality after harvest. Overall, these formulations limit microbial spoilage and reduce respiration and transpiration rates, slow down color changes, and decrease the degradation of ascorbic acid, phenolic compounds, and anthocyanins.^30^

Several studies have shown that layer‐by‐layer coatings provide greater preservation efficacy than monolayer systems by enhancing barrier properties, delaying ripening, and reducing microbial decay across different fruit crops.^11^ However, previous research has largely focused on external quality parameters, while the underlying mechanisms through which bilayer coatings modulate oxidative stress remain poorly understood.

In this context, the study presented here aimed to evaluate the effectiveness of a CMC–alginate bilayer edible coating in preserving strawberry quality during refrigerated storage through an integrated assessment of physicochemical, biochemical, and multivariate physiological responses.

## MATERIALS AND METHODS

### Strawberry samples and postharvest treatment

Strawberries of ‘Rossetta’ cultivar were collected at the Cooperativa Sole (40.9605736 N, 14.1482696° E, Parete, CE, Italy) and transferred to the pomology laboratory of CREA‐Research Centre for Olive, Fruit and Citrus Crops. They were selected based on uniformity of size, color, and shape, eliminating any fruit with physical or mechanical defects.

The fruits were divided into two batches. The first batch was immersed for 2 min in a solution of calcium chloride (2% w/v), followed by CMC (1% w/v), then sodium alginate (1% w/v), and finally a solution of calcium chloride (2% w/v) (coated sample). Immersion in each solution was alternated with a drying period at room temperature (about 1 h). Strawberries in the second batch underwent the same treatment as the treated strawberries, but the immersion solutions were replaced with sterile distilled water (uncoated sample).

Both coated and uncoated samples were prepared by placing approximately 100 g of strawberries (about four fruits) into individual polyethylene terephthalate boxes with lids. At each sampling time (T0, T1, T3, T6, and T8), three independent boxes were used for each treatment (three control and three coated boxes), corresponding to six boxes and 24 fruits per sampling time. Thus, the experimental design comprised a total of 30 boxes (15 control and 15 coated), corresponding to 120 strawberries. Each box represented one biological replicate. Strawberries were then stored in a laboratory refrigerator at 4 °C and 95% relative humidity for 8 days. All analyses were performed on days 0, 1, 3, 6, and 8 (Fig. 1).

**Figure 1:**
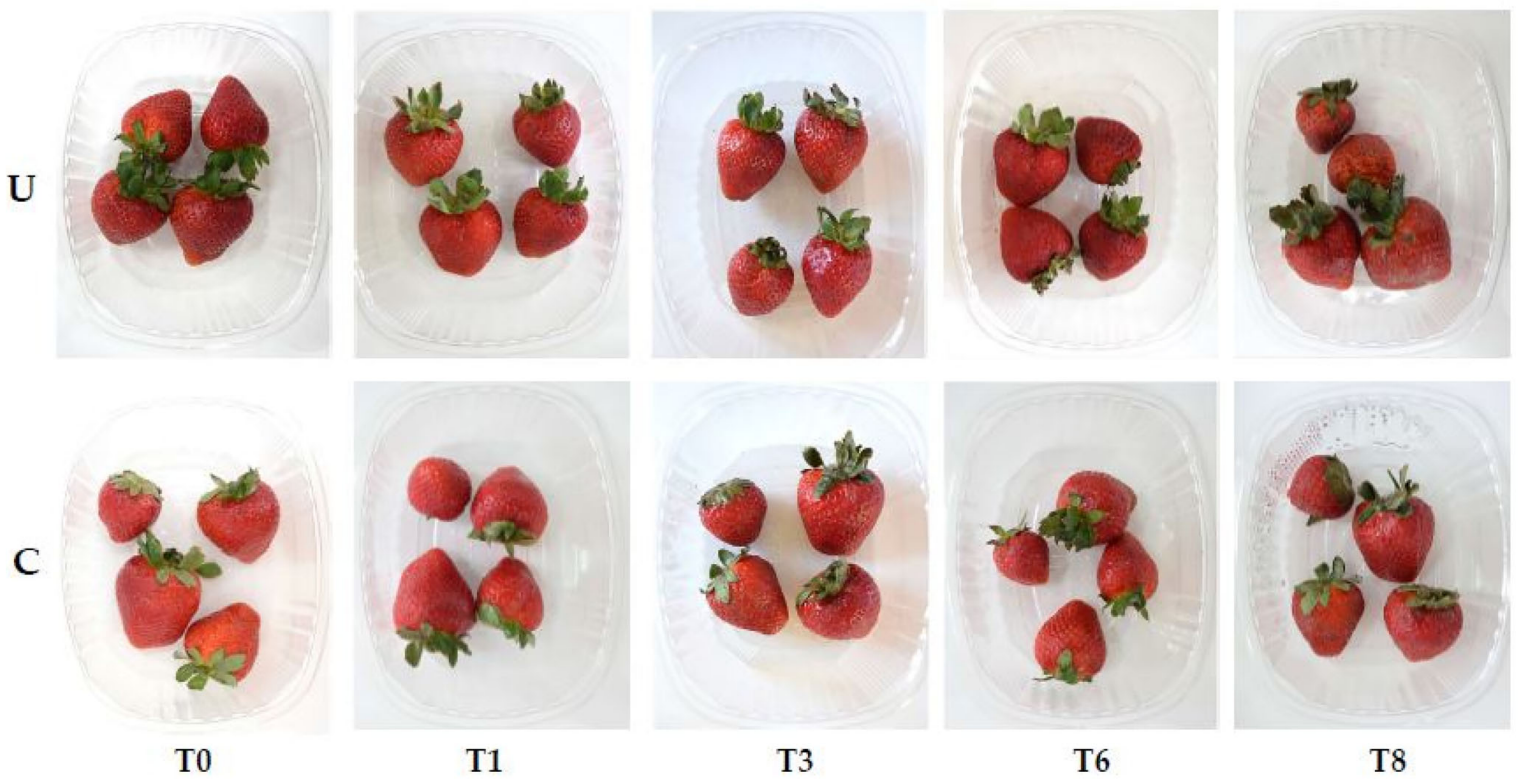
Strawberry samples during cold storage at 4 °C for 8 days. Control (C) and treated (T) fruits placed in individual polyethylene terephthalate containers at different sampling times (T0, T1, T3, T6 and T8).

### Analytical determinations

#### Chemical–physical analysis

Weight loss was calculated according to the weight of each sample before and after storage, and expressed as a percentage of weight loss relative to the initial weight. The firmness of the pulp was determined using a penetrometer (TR snc, Forlì, Italy) with an 8 mm probe and expressed in newtons. Skin color was assessed using a Minolta colorimeter (CR5, Minolta Camera Co., Osaka, Japan) utilizing the CIELAB color space (L*, lightness; a*, green to red; b*, blue to yellow). The total soluble solids content (TSS, °Brix) was determined using a digital refractometer (Sinergica Soluzioni, DBR35, Pescara, Italy). Titratable acidity (TA) was determined by titration with 0.1 N NaOH until pH 8.1, and expressed as grams of citric acid per liter. The pH was determined using a digital pH meter.

#### Determination of bioactive compounds and antioxidant activity

For the extraction of bioactive compounds, 1 g of fruit was weighed and homogenized in 5 mL of 80% (v/v) methanol. The sample was then placed on a shaking plate overnight for 24 h in the dark. Subsequently, the sample was centrifuged at 13 000 rpm for 15 min at 4 °C. The supernatant was collected and stored at 4 °C for subsequent analyses.

Total polyphenol content was determined using the Folin–Ciocalteu colorimetric method.^31^ Polyphenols were quantified using a standard curve prepared with known concentrations of gallic acid. Values are expressed as milligrams of gallic acid equivalents (GAE) per 100 g of fresh weight (FW).

The flavonoid assay was conducted using the aluminium chloride colorimetric method.^32^ The total flavonoid content (TFC) was quantified using a standard curve prepared with known concentrations of catechin and expressed as milligrams of catechin equivalent (CE) per 100 g of FW.

The anthocyanin assay was performed using the differential pH spectrophotometric method.^33^ The anthocyanin content was determined using a standard curve at known concentrations of cyanidin‐3‐glucoside. Results are expressed as cyanidin‐3‐glucoside equivalents (CGE) per 100 g of FW. Absorbance was measured at 520 and 700 nm.

The total antioxidant activity was measured using 2,2′‐azinobis(3‐ethylbenzothiazoline‐6‐sulfonic acid) (ABTS). The assay was conducted as described by Ferrara *et al*.^34^ The test was performed in a final volume of 1 mL. The percentage decrease in ABTS concentration was calculated relative to the initial value after a 6 min reaction. A dose–response curve was constructed to determine the concentration of the product required to achieve 50% of the initial concentration (IC50). Trolox was used as a reference antioxidant, and antioxidant activity is expressed as Trolox equivalents (TE). TE represent the ratio between the IC50 of Trolox (μmol) and the IC50 of the sample (g^−1^ FW).

#### Enzymatic activity

Strawberry samples were ground into a powder using liquid nitrogen. The pulverized material was then resuspended in extraction buffer (1:5 m/v) containing 50 mmol L^−1^ potassium phosphate buffer (pH 7.8), 1 mmol L^−1^ sodium‐EDTA (pH 7.0), and 2% (w/v) polyvinylpolypyrrolidone (PVPP). For the ascorbate peroxidase (APX) assay only, 0.35 mmol L^−1^ hydrogen peroxide (H_2_O_2_) and 0.33 mmol L^−1^ ascorbic acid were added to the extraction solution.

The samples were homogenized in a mortar, transferred to 2 mL Eppendorf tubes kept on ice, and subsequently placed in a Thermomixer (Eppendorf) for agitation. They were then centrifuged at 14000 × *g* for 15 min at 4 °C. The supernatant was transferred to a new Eppendorf tube and used for enzyme activity measurements. The protein content in all crude enzyme extracts was estimated using the Bradford assay,^35^ with bovine serum albumin as the standard.

The activity of superoxide dismutase (SOD; EC 1.15.1.1) was determined by evaluating the inhibition of the photochemical reduction of nitroblue tetrazolium (NBT) in the presence of riboflavin, according to the protocol described by García‐Limones *et al*.,^36^ with minor modifications. The reaction mixture contained 100 μL of extract, 500 mmol L^−1^ potassium phosphate buffer (pH 7.8), 0.1 mmol L^−1^ sodium‐EDTA (pH 7), 13 mmol L^−1^ methionine, 75 μmol L^−1^ NBT, and 2 μmol L^−1^ riboflavin. Once the assay samples were prepared, the tubes, along with a blank, were exposed to light for 15 min, while two other blanks were kept in the dark. Absorbance was then measured spectrophotometrically at 560 nm. Enzyme activity was expressed in U g^−1^ FW.

Catalase (CAT; EC 1.11.1.6) activity was assayed according to the method of Fernandez‐Trujillo *et al*.,^37^ with some modifications. The reaction medium contained 200 μL of extract, 50 mmol L^−1^ potassium phosphate buffer (pH 7), and 20 mmol L^−1^ H_2_O_2_, for a final volume of 1.5 mL. CAT activity commenced with the addition of the substrate (H_2_O_2_) to the reaction mixture and was detected spectrophotometrically by recording the decrease in H_2_O_2_ content at 240 nm for a maximum of 200 s. CAT activity is expressed as μmol of H_2_O_2_ g^−1^ FW.

The activity of APX (EC 1.11.1.11) was determined according to Rao *et al*.^38^ The reaction mixture consisted of 100 mmol L^−1^ potassium phosphate buffer (pH 7), 0.33 mmol L^−1^ ascorbic acid, 0.35 mmol L^−1^ H_2_O_2_, 0.66 mmol L^−1^ sodium‐EDTA (pH 7), and 100 μL of enzyme extract in a final volume of 1.5 mL. The APX‐catalyzed reaction began with the addition of H_2_O_2_ and ascorbic acid to the reaction mixture and was detected spectrophotometrically by recording the decrease in ascorbate content at 290 nm for a maximum of 100 s. APX activity is expressed as μmol of ascorbate g^−1^ FW.

The activity of polyphenol oxidase (PPO; EC 1.30.3.1) was determined according to the protocol by Cao *et al*.,^39^ with slight modifications. First, 2.5 g of pulverized material was homogenized with 5 mL of 200 mmol L^−1^ sodium phosphate buffer (pH 6.5) containing 0.100 g of PVPP. The homogenate was incubated on ice with agitation for 1 h and then centrifuged at 12 500 rpm for 10 min at 4 °C. The recovered supernatant was then added to a reaction mixture consisting of 1.4 mL of 0.07 mol L^−1^ catechol dissolved in 0.5 mol L^−1^ sodium dihydrogen phosphate buffer (pH 6.5). Catechol oxidation, and thus PPO activity, was detected spectrophotometrically at 398 nm and expressed as nmol of catechol g^−1^ FW. Lipoxygenase (LOX) activity was quantified according to the method of Pérez *et al*.,^40^ with minor modifications. The enzyme was extracted by resuspending 1 g of pulverized material with 3 mL of extraction buffer (500 mmol L^−1^ potassium phosphate buffer (pH 7.8), 1 mmol L^−1^ sodium‐EDTA (pH 7), and 2% (w/w) PVPP). The homogenate was then centrifuged at 12 500 rpm for 10 min at 4 °C. The recovered supernatant was added to a reaction mixture composed of 1.4 mL of 100 mmol L^−1^ sodium phosphate buffer (pH 6), 50 μL of 5 mmol L^−1^ linoleic acid sodium salt, and 50 μL of enzyme extract in a final volume of 1.5 mL. LOX activity was detected spectrophotometrically based on hydroperoxide formation and the consequent increase in absorbance at 234 nm. LOX activity was expressed as nmol hydroperoxides min^−1^ g^−1^ FW.

The malondialdehyde (MDA) content was evaluated according to the method of Peever and Higgins,^41^ with modifications. A total of 1 g of fruit pulp, pulverized in liquid nitrogen, was homogenized with 10 mL of extraction buffer (10% trichloroacetic acid, 0.25% thiobarbituric acid in 0.25 N HCl). Samples were heated at 95 °C for 30 min and subsequently cooled on ice, except for the blank, which was prepared using the same method without heating. Subsequently, all samples were centrifuged at 10 000 × *g* for 10 min at 4 °C. A 1 mL aliquot of the supernatant was used for the spectrophotometric determination of MDA at wavelengths of 432, 532, and 600 nm. MDA content was expressed as μmol (100 g)^−1^ FW.

### Statistical analysis

All the analyses were conducted in triplicate on three biological replicates. Analytical data were subjected to the Bartlett and Shapiro–Wilk tests to verify normality and homogeneity of variances. Once these prerequisites were verified, data were analyzed using two‐way analysis of variance (ANOVA), considering both storage time (T0, T1, T3, T6, T8) and treatment (control *versus* coating), and a *post hoc* Duncan test for pairwise comparisons with *P* ≤ 0.05 (IBM SPSS Statistics for Windows, Version 27.0). ANOVA–simultaneous component analysis (ASCA) was performed to assess the effects of storage time, treatment, and their interaction on the multivariate physiological/biochemical response of strawberries. The data matrix was decomposed into effect matrices according to two‐way factorial design, and principal component analysis was applied to each effect matrix to visualize sample distribution and variable contributions. The significance of each effect was assessed by residual permutation testing according to Zwanenburg *et al*.^42^ (*P* < 0.05). Finally, Pearson correlation coefficients were calculated among all quantitative variables using pairwise complete observations. The resulting correlation matrix was visualized as a clustered heatmap by hierarchical clustering using complete linkage and a correlation‐based distance metric. All analyses were performed in R (version 4.5.2).

## RESULTS AND DISCUSSION

### Physicochemical parameters

Weight loss increased during cold storage in both coated and uncoated strawberries, with significant differences between treatments from day 3 onwards (Table 1). After 8 days at 4 °C, control fruit reached a weight loss of 4.62 ± 0.13%, whereas coated strawberries showed a significantly lower value (approximately 25% lower). This behavior is consistent with the role of edible coating as a semipermeable barrier, which limits transpiration and gas exchange, as previously reported.^43^ Fruit firmness decreased over time in both treatments, reflecting ongoing postharvest ripening and softening. However, coated strawberries retained higher firmness than controls at the end of storage. The application of the alginate–CMC coating therefore mitigated flesh softening, likely by slowing down moisture loss and the metabolic processes associated with cell wall degradation, in agreement with previous studies on chitosan‐ and polysaccharide‐based coatings applied to strawberries.^44^ TSS increased during storage in both coated and uncoated samples, but the rise was more pronounced in control fruit. This pattern suggests a concentration effect due to more intense weight loss in uncoated fruit, whereas the coating, reducing transpiration and respiratory activity, limited both dehydration and hydrolysis of polysaccharides into soluble sugars.^11^, ^45^ TA gradually decreased, with a more marked reduction in uncoated samples. This trend is consistent with the metabolic consumption and degradation of organic acids during respiration and senescence.^26^ In contrast, pH values remained relatively stable and did not differ significantly between treatments. Overall, the combined evolution of TSS, TA and pH confirms that the edible coating contributed to slowing down postharvest ripening.

**Table 1:** Effect of edible coating on weight loss, firmness, total soluble solids (TSS), pH, and titratable acidity (TA expressed as citric acid g L^−1^) in strawberries during 8 days of cold storage at 4 °C

Sampling time (days)	Treatment	Weight loss (%)	Firmness (N)	TSS (°Brix)	pH	TA (g L^−1^)
0	C	0.00 ± 0.00 ^h^	5.10 ± 0.68 ^ab^	5.63 ± 0.76 ^b^	3.55 ± 0.11 ^ab^	5.12 ± 0.32 ^a^
	T	0.00 ± 0.00 ^h^	5.19 ± 0.59 ^a^	5.57 ± 0.71^b^	3.56 ± 0.08 ^ab^	5.10 ± 0.22 ^a^
1	C	0.67 ± 0.06 ^g^	4.08 ± 1.11 ^cd^	6.10 ± 0.60 ^ab^	3.67 ± 0.05 ^a^	5.01 ± 0.49 ^a^
	T	0.53 ± 0.04 ^g^	4.83 ± 0.89 ^ab^	5.73 ± 0.68 ^b^	3.55 ± 0.68 ^ab^	4.97 ± 0.16 ^a^
3	C	1.92 ± 0.04 ^e^	4.04 ± 0.60 ^cd^	6.00 ± 0.75 ^ab^	3.59 ± 0.06 ^ab^	4.91 ± 0.37 ^a^
	T	1.41 ± 0.04 ^f^	4.86 ± 0.79 ^ab^	5.80 ± 0.17 ^b^	3.58 ± 0.02 ^ab^	4.84 ± 0.26 ^a^
6	C	3.21 ± 0.07 ^c^	3.62 ± 0.82 ^d^	6.57 ± 0.40 ^ab^	3.60 ± 0.04 ^ab^	4.69 ± 0.49 ^a^
	T	2.44 ± 0.22 ^d^	4.85 ± 0.80 ^ab^	6.13 ± 0.51 ^ab^	3.60 ± 0.06 ^ab^	4.91 ± 0.74 ^a^
8	C	4.62 ± 0.13 ^a^	3.69 ± 0.99 ^d^	7.00 ± 0.26 ^a^	3.50 ± 0.15 ^b^	4.59 ± 0.49 ^a^
	T	3.43 ± 0.13 ^b^	4.52 ± 0.99 ^bc^	6.30 ± 0.42 ^ab^	3.64 ± 0.09 ^ab^	4.80 ± 0.55 ^a^

Data are expressed as mean ± standard deviation (*n* = 3). Different letters within columns indicate significant differences among sampling times and treatments according to two‐way ANOVA and Duncan *post hoc* multiple comparison tests (*P* < 0.05). C, control; T, treated.

Color is a key visual attribute in consumer perception of strawberry quality, and minimizing color loss during storage contributes to reducing waste and associated economic losses.^46^ The evolution of color parameters (L*, a*, b*) during storage is reported in Table 2. A progressive decline in L* values was observed in coated and uncoated strawberries, indicating a general darkening of the fruit surface. However, the reduction in brightness was less pronounced in coated strawberries, which consistently retained higher L* values than the control at the end of storage. This suggests that the coating slowed down surface browning. Similarly, the a* (red–green) and b* (yellow–blue) chromaticity coordinates decreased during storage, reflecting the degradation of anthocyanins and other pigments. Coated fruit exhibited significantly lower a* and b* values than the control samples at several time points, a trend also reported in strawberries coated with chitosan enriched with hawthorn extract^47^ and with pectin‐based coatings.^44^ The lower chromatic values in coated samples may be linked to reduced metabolic activity and pigment oxidation due to the semipermeable barrier formed by the coating.

**Table 2:** Effect of edible coating on lightness (L*), green to red (a*), blue to yellow (b*) of strawberries during 8 days of cold storage at 4 °C

Sampling time (days)	Treatment	L*	a*	b*
0	C	34.12 ± 1.61 ^a^	37.35 ± 2.19 ^a^	21.88 ± 2.04 ^a^
	T	27.96 ± 1.40 ^e^	34.98 ± 1.17 ^b^	20.86 ± 1.47 ^a^
1	C	31.54 ± 1.54 ^b^	36.55 ± 1.71 ^a^	21.69 ± 1.52 ^a^
	T	31.14 ± 1.21 ^b^	37.29 ± 1.10 ^a^	21.88 ± 1.55 ^a^
3	C	27.80 ± 1.70 ^ef^	32.04 ± 1.66 ^c^	19.04 ± 1.64 ^b^
	T	28.79 ± 1.40 ^de^	31.57 ± 1.55 ^c^	18.29 ± 1.38 ^bc^
6	C	27.63 ± 1.53 ^ef^	31.51 ± 1.86 ^c^	18.08 ± 1.73 ^bc^
	T	30.56 ± 1.90 ^bc^	29.79 ± 1.87 ^d^	16.36 ± 1.69 ^de^
8	C	26.57 ± 1.49 ^f^	31.35 ± 1.77 ^c^	17.54 ± 1.84 ^cd^
	T	29.43 ± 0.72 ^cd^	29.15 ± 1.74 ^d^	15.93 ± 1.40 ^e^

Data are expressed as mean ± standard deviation (*n* = 3). Different letters within columns indicate significant differences among sampling times and treatments according to two‐way ANOVA and Duncan *post hoc* multiple comparison tests (*P* < 0.05). C, control; T, treated.

### Bioactive compounds and antioxidant activity

Strawberries are rich in bioactive molecules such as vitamins, fibers, anthocyanins, flavonoids, and polyphenols, which contribute to their nutritional value. However, these compounds are highly sensitive to postharvest handling, and their retention strongly depends on storage conditions.^48^ Polyphenols and anthocyanins contribute to the characteristic red color and are known for their protective effects against free radical damage and potential health‐promoting properties.^49^, ^50^, ^51^

The evolution of total polyphenol content (TPC), flavonoids, anthocyanins, and antioxidant activity during cold storage is presented in Table 3. Overall, a general increase of bioactive compounds was observed in both treatments, although coated strawberries exhibited higher values than the control from day 1 onwards. At day 6, when the highest content was recorded, coated samples significantly exceeded the respective values measured in the control fruit. A similar pattern was observed for antioxidant activity. The observed increase in phenolic compounds and antioxidant capacity in coated fruit may be associated with metabolic responses triggered by cold storage and with a protective effect of the edible coating. Several studies have demonstrated that the application of edible coatings postharvest can activate fruit defense responses, particularly the synthesis of phenolic compounds.^11^, ^52^ Moreover, coatings can act as semipermeable barriers, reducing gas exchange and moisture loss and contributing to a modified internal atmosphere. This can slow oxidative processes, delay pigment degradation, and preserve cellular integrity. Also, coatings may promote the redistribution and accumulation of phenolic metabolites within fruit tissues during storage, as previously reported for chitosan‐based treatments.^53^

**Table 3:** Effect of edible coating on total polyphenols (TPC, mg GAE (100 g)^−1^ FW), flavonoids (mg CA (100 g)^−1^ FW), anthocyanins (mg (100 g)^−1^ FW), and antioxidant activity (ABTS assay, μmol g^−1^ FW) of strawberries during 8 days of cold storage at 4 °C

Sampling time (days)	Treatment	TPC	Flavonoids	Anthocyanins	ABTS
0	C	156.75 ± 2.12 ^de^	33.07 ± 0.62 ^c^	18.06 ± 0.29 ^e^	31.40 ± 0.46 ^cd^
	T	153.58 ± 4.01 ^de^	32.06 ± 0.39 ^c^	19.25 ± 0.77 ^e^	30.80 ± 0.45 ^cd^
1	C	153.48 ± 16.97 ^de^	29.35 ± 4.13 ^c^	29.90 ± 2.04 ^d^	29.75 ± 2.27 ^cd^
	T	194.66 ± 19.67 ^b^	43.05 ± 7.63 ^b^	19.06 ± 1.89 ^e^	36.18 ± 3.08 ^b^
3	C	134.56 ± 1.05 ^f^	28.70 ± 0.59 ^c^	35.68 ± 0.61 ^b^	26.10 ± 0.27 ^e^
	T	142.21 ± 10.57 ^ef^	33.20 ± 0.90 ^c^	30.91 ± 1.36 ^cd^	29.39 ± 0.20 ^d^
6	C	177.88 ± 0.95 ^c^	39.10 ± 0.39 ^b^	38.74 ± 1.30 ^a^	36.17 ± 0.61 ^b^
	T	221.07 ± 3.95 ^a^	53.31 ± 0.83 ^a^	33.25 ± 1.09 ^bc^	45.02 ± 1.64 ^a^
8	C	162.79 ± 3.35 ^cd^	30.92 ± 0.92 ^c^	32.74 ± 0.16 ^c^	32.28 ± 0.49 ^c^
	T	170.08 ± 3.16 ^cd^	31.18 ± 2.79 ^c^	39.30 ± 3.31 ^a^	35.06 ± 1.06 ^b^

Data are expressed as mean ± standard deviation (*n* = 3). Different letters within columns indicate significant differences among sampling times and treatments according to two‐way ANOVA and Duncan *post hoc* multiple comparison tests (*P* < 0.05). C, control; T, treated.

### Antioxidant enzymatic activity

Oxidative stress induced by low temperatures and/or senescence phenomena occurring during fruit storage leads to an increase in the levels of reactive oxygen species (ROS). ROS are toxic molecules present in cells that are efficiently scavenged by nonenzymatic antioxidants (*α*‐tocopherol, *β*‐carotene, phenolic compounds, ascorbate, glutathione)^54^, ^55^, ^56^ as well as by enzymatic antioxidants.^57^

The enzymatic antioxidant system includes SOD, a group of metalloenzymes that catalyze the dismutation of the superoxide radical (O_2_^−^) into hydrogen peroxide (H_2_O_2_) and molecular oxygen. The H_2_O_2_ produced is subsequently eliminated through the catalytic activity of other enzymes, such as CAT, which converts H_2_O_2_ into water and molecular oxygen; APX, which consumes H_2_O_2_ converting it into dehydroascorbate and water; and guaiacol‐dependent peroxidase, which transforms H_2_O_2_ into tetraguaiacol and water.

SOD activity increased during cold storage, with higher values observed in treated fruits. These results suggest that the coating may induce an enhancement of SOD activity, thereby strengthening the ROS scavenging potential in strawberries (Fig. 2).

**Figure 2:**
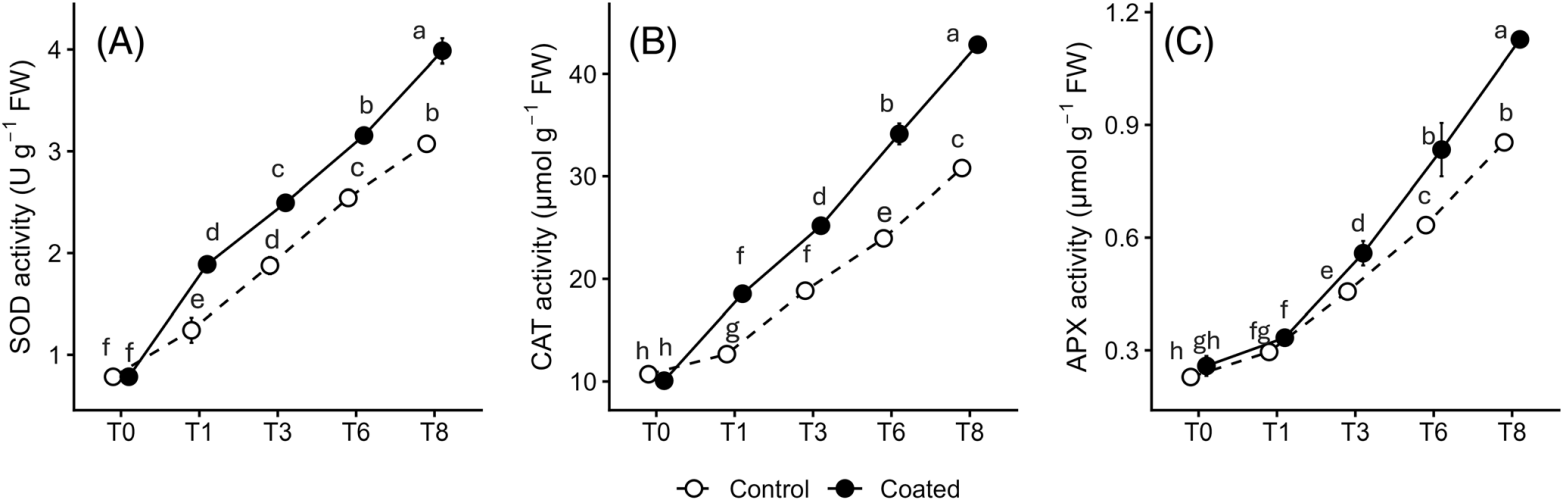
Effect of CMC–alginate bilayer edible coating on antioxidant enzyme activities in strawberries during 8 days of refrigerated storage at 4 °C: (A) SOD, (B) CAT, and (C) APX. Values are expressed as mean ± standard deviation (*n* = 3). Different letters indicate significant differences among sampling times and treatments according to two‐way ANOVA followed by Duncan's multiple range test (*P* < 0.05). Control, uncoated strawberries; coated, CMC–alginate‐coated strawberries.

As previously described, CAT catalyzes the decomposition of hydrogen peroxide into water and oxygen.^58^ This enzyme plays a fundamental role in counteracting oxidative stress, as H_2_O_2_ is extremely toxic and can easily diffuse across biological membranes.^59^ During cold storage, CAT activity tended to increase in all the samples, but more rapidly in treated fruits. These results demonstrate that the treatment slows down some of the biochemical reactions associated with senescence processes in strawberries, as also reported in previous studies.^60^, ^61^ An alternative pathway for H_2_O_2_ removal involves peroxidases, which reduce H_2_O_2_ to water using a reducing agent. Peroxidases have been associated with numerous physiological processes, including leaf and flower abscission, ageing and senescence, cold tolerance, dormancy, fruit development and ripening, germination, and hypersensitive resistance responses.^59^

APX is generally located in chloroplasts, but isoforms are also present in microsomes and peroxisomes, as well as isoenzymes in the cytosol and apoplast.^57^ Wang *et al*.^62^ also investigated the antioxidant properties of a membrane‐bound APX located in peroxisomes. APX is a highly labile enzyme compared to other peroxidases and it is inactivated at high H_2_O_2_ concentrations. It is involved in cellular detoxification processes by using ascorbate as an electron donor, consuming hydrogen peroxide and converting it into dehydroascorbate and water. During the 8 days of cold storage, APX activity increased in all samples, indicating that the rise in enzymatic activity is directly related to increasing stress levels and that the treatment delays the onset of senescence‐related effects, as higher APX activity values were recorded in treated fruits compared to controls. The increase in APX activity has also been associated with the acquisition of cold tolerance in several fruits during cold storage.^63^, ^64^, ^65^

### Fruit browning and oxidative damage to cell membranes

The onset of chilling injury primarily affects the cell membrane, which represents the first cellular compartment involved in cold‐induced damage.^66^ Fruit tolerance to low temperatures is therefore strongly dependent on the preservation of membrane integrity.^67^ Membrane degradation under chilling conditions is mainly associated with oxidative processes leading to the formation of free radicals, as well as with the activity of specific enzymes such as LOX. LOX catalyzes the oxidation of polyunsaturated fatty acids, producing conjugated hydroperoxides and initiating the LOX pathway, which plays a key role in plant responses to stress and injury.^68^

Oxidative stress is considered one of the earliest events occurring during chilling injury, resulting from an imbalance between the production of ROS and their scavenging by antioxidant systems. Disruption of this redox equilibrium leads to the accumulation of toxic compounds, including free radicals and peroxides.^69^, ^70^, ^71^ A well‐established marker of membrane lipid peroxidation is MDA, whose accumulation is commonly used as an indirect indicator of membrane integrity loss.^69^

Membrane deterioration and oxidative stress are also closely linked to senescence‐related processes occurring during cold storage, including tissue browning. This phenomenon is partly driven by PPO, which catalyzes the *o*‐hydroxylation of monophenols and the oxidation of diphenols to *o*‐quinones, using molecular oxygen.^72^ Under normal physiological conditions, PPO and its phenolic substrates are compartmentalized: PPO is located in the thylakoid lumen of plastids, whereas phenolic compounds are stored in vacuoles. Their interaction occurs only when membrane integrity is compromised, allowing enzyme–substrate contact following cellular damage. The resulting quinones subsequently polymerize, causing visible discoloration of tissues from yellow to brown.

In addition to PPO activity, changes in anthocyanin content contribute to browning development. Anthocyanin degradation is associated with coupled oxidation reactions mediated by PPO in the presence of other phenolic compounds.^73^, ^74^, ^75^ More generally, pigment degradation in fruits is exacerbated by physiological stress conditions such as water loss, which accelerates senescence, promotes membrane disruption, and facilitates the release of oxidative enzymes and their substrates, thereby enhancing the oxidation of cellular components.^76^

In agreement with these mechanisms, the experimental results showed that PPO activity increased in all samples during cold storage (Fig. 3). However, the coating slowed down the increase in PPO activity, in line with previous findings.^60^, ^77^ Similarly, LOX activity, which plays a central role in lipid peroxidation and membrane damage,^78^, ^79^ increased progressively during cold storage. This trend is consistent with the known effect of cold stress in promoting LOX‐mediated oxidation of fatty acids.^78^ Notably, treated fruits exhibited significantly lower LOX activity. Comparable results have been reported in watermelon, where chilling injury development was associated with increased LOX activity under cold stress conditions.^80^

**Figure 3:**
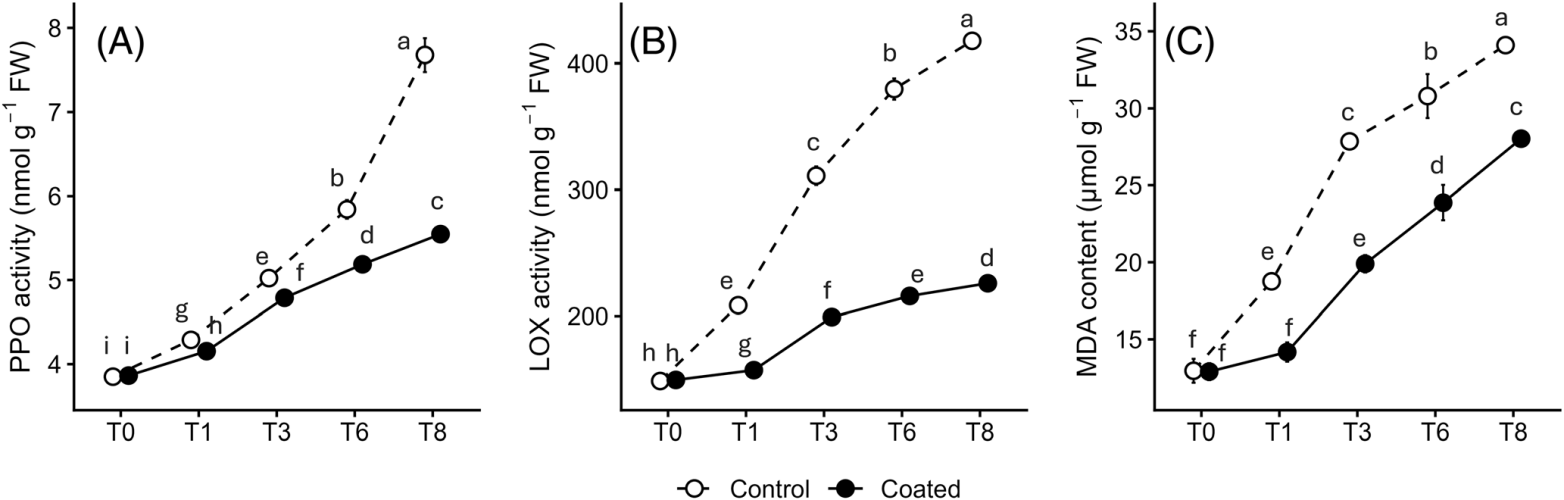
Effect of CMC–alginate bilayer edible coating on oxidative deterioration‐related parameters in strawberries during 8 days of refrigerated storage at 4 °C: (A) PPO activity, (B) LOX activity, and (C) MDA content. Values are expressed as mean ± standard deviation (*n* = 3). Different letters indicate significant differences among sampling times and treatments according to two‐way ANOVA followed by Duncan's multiple range test (*P* < 0.05). Control, uncoated strawberries; coated, CMC–alginate‐coated strawberries.

Consistently, MDA content increased with storage time, confirming the progression of lipid peroxidation. Untreated fruits showed significantly higher MDA levels than treated ones (Fig. 3). Since MDA accumulation is closely correlated with oxidative damage under stress conditions,^80^ these results further indicate that the treatment mitigated membrane deterioration.

The effectiveness of the coating in preserving fruit quality can be attributed to its ability to act as a barrier to oxygen diffusion, thereby limiting oxidative reactions responsible for membrane lipid peroxidation and browning development.^61^, ^81^ Overall, the treatment contributed to maintaining membrane integrity and reducing fruit deterioration during cold storage.

### Multivariate assessment of physiological responses during cold storage

Since numerous quality traits evolved simultaneously during storage, ASCA was applied to obtain an integrated interpretation of the multivariate physiological response. The permutation test confirmed that storage time, treatment, and their interaction all exerted significant effects on the multivariate response of strawberries (*P* = 0.001; Table 4). Among these, storage time represented by far the dominant source of variation, explaining 61.99% of the total sum of squares, whereas treatment and the treatment × time interaction accounted for 8.52% and 10.22%, respectively. The biplot plot associated with the storage time effect (Fig. 4(A)) showed a clear temporal progression along PC1, which explained 63.2% of the variability. Samples followed an ordered trajectory from T0 to T8, indicating that storage time was the principal driver of the physiological and biochemical evolution of strawberry fruit. Weight loss, oxidative stress‐related parameters (PPO, LOX, and MDA), and antioxidant enzymes (SOD, CAT, and APX) were positively associated with the positive side of PC1, corresponding to the advanced stages of storage. On the other hand, firmness, TA, and color were mainly associated with freshly harvested fruit. In contrast, antioxidant‐related variables (TPC, TFC, and ABTS) were predominantly associated with PC2, suggesting a distinct temporal evolution compared with the other quality parameters.

**Table 4:** Results of ASCA

Effect	SSQ	Explained variance (%)	Permutation test (*P*‐value)
Storage time	359.57	61.99	0.001
Treatment	49.43	8.52	0.001
Storage time × treatment	59.25	10.22	0.001

Sum of squares (SSQ), percentage of explained variance, and permutation test (*P*‐values) for the effects of storage time, treatment, and their interaction on the multivariate physiological and biochemical dataset.

**Figure 4:**
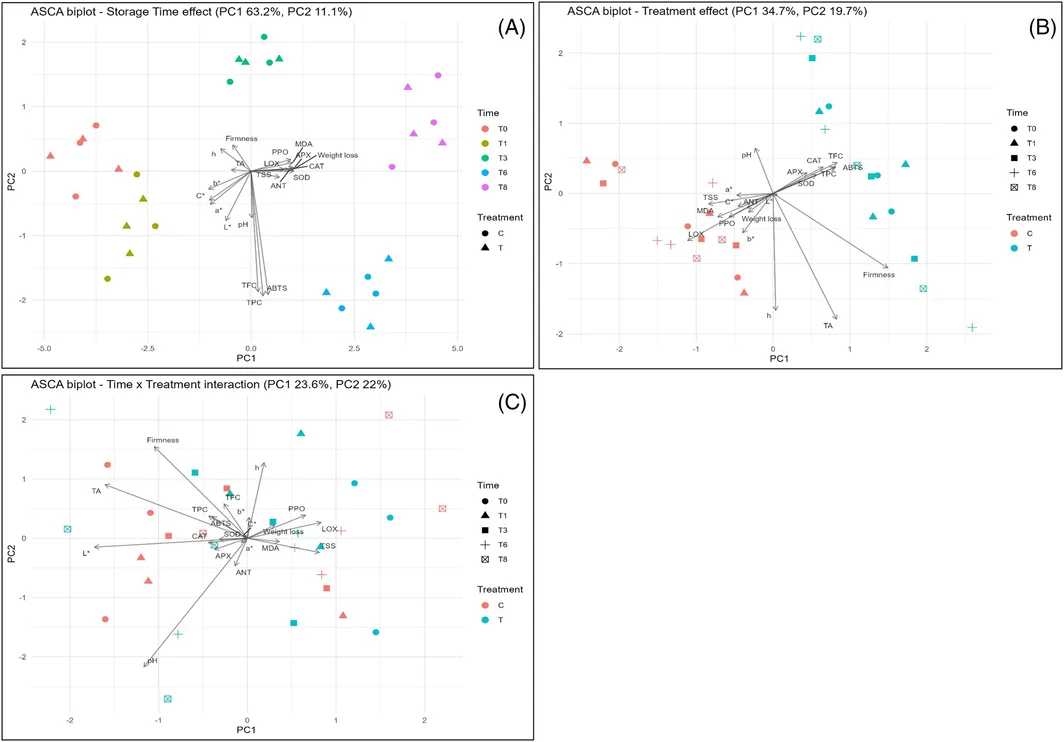
ASCA score and loading plots describing the multivariate effects of (A) storage time, (B) treatment, and (C) storage time × treatment interaction on the physicochemical, biochemical, and physiological parameters of strawberries during refrigerated storage. Percentages reported for PC1 and PC2 indicate the variance explained by each principal component within the corresponding ASCA effect matrix. C, control; T, coated strawberries; T0–T8, storage time.

The treatment effect (Fig. 4(B)) accounted for a smaller but significant proportion of the overall variability. PC1 (34.7%) discriminated coated and control strawberries, with coated fruit mainly associated with higher antioxidant activity (TPC, TFC, and ABTS), greater firmness retention, and increased activities of SOD, CAT, and APX. Conversely, control fruit showed a closer association with oxidative damage markers (LOX, PPO, and MDA), as well as higher weight loss and more pronounced color changes, indicating a greater progression of senescence during storage.

The interaction explained a lower proportion of the total variability (10.22%) but still it was significant (*P* = 0.001). The interaction plot (Fig. 4(C)) indicates that the contribution of oxidative stress markers and antioxidant enzymes varied according to both storage time and coating treatment, suggesting that the edible coating modulated the temporal evolution of strawberry physiology rather than producing a constant treatment effect.

To further investigate the relationships among the measured variables, Pearson correlation analysis coupled with hierarchical clustering was performed (Fig. 5) which revealed the presence of distinct groups of highly correlated variables, reflecting coordinated physiological responses occurring during strawberry storage. The heatmap highlighted a tightly connected subgroup composed of SOD, CAT, and APX, which showed strong positive correlations with one another (*r* = 0.97–0.99), indicating the coordinated activation of the enzymatic antioxidant defense system.^57^ These enzymes were also positively associated with anthocyanins, TSS, weight loss, and markers of oxidative deterioration (PPO, LOX, and MDA), suggesting that their induction occurred as storage progressed and oxidative stress increased. A second cluster grouped PPO, LOX, MDA, and weight loss, which exhibited strong positive correlations (*r* = 0.84–0.95). These variables were negatively correlated with firmness and color parameters, confirming the close association between membrane lipid peroxidation, enzymatic browning, tissue softening, and visual quality loss during postharvest storage. These findings are consistent with the physiological role of LOX and PPO in membrane degradation and browning development, as well as with MDA accumulation as an indicator of lipid peroxidation.^60^, ^78^, ^80^

**Figure 5:**
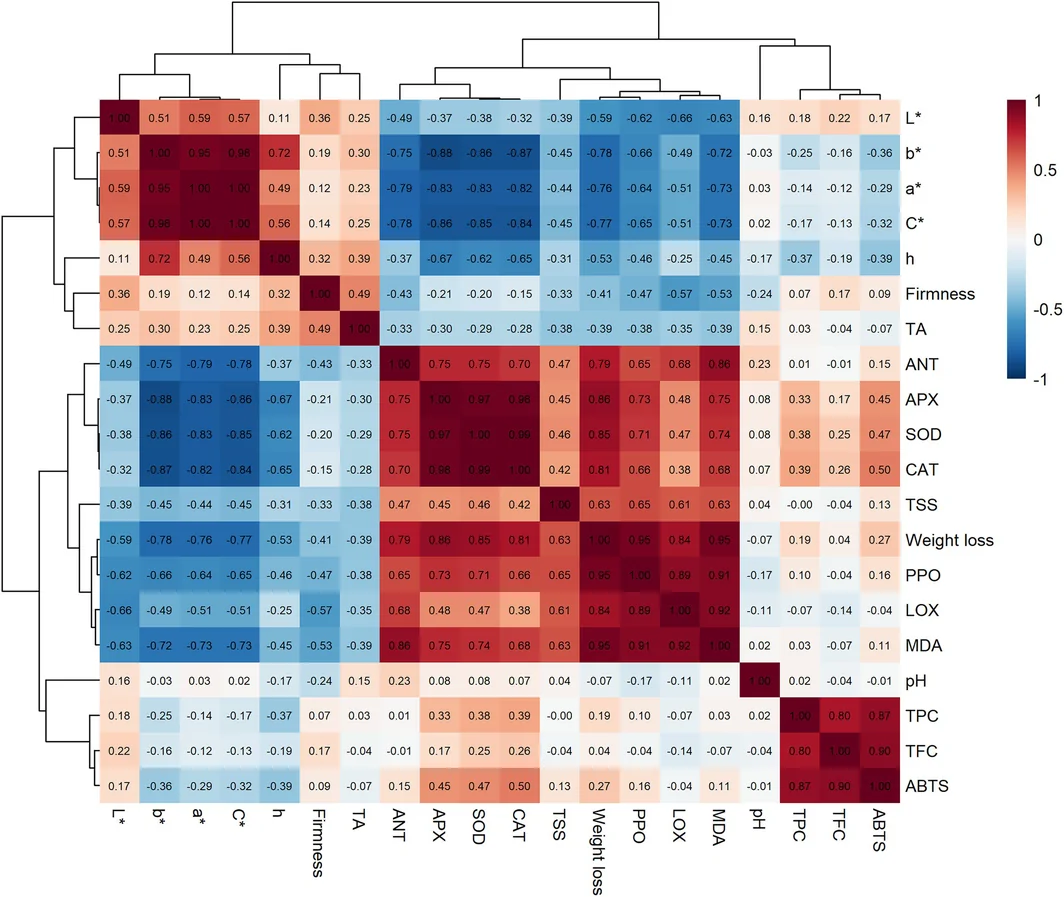
Pearson correlation heatmap and hierarchical clustering of physicochemical, biochemical, and physiological parameters measured in strawberries during refrigerated storage. Cell values represent Pearson correlation coefficients (*r*), with positive and negative correlations indicated by red and blue, respectively. Dendrograms represent hierarchical clustering of variables based on their correlation patterns. TPC, total phenolic content; TFC, total flavonoid content; ANT, anthocyanins; SOD, superoxide dismutase; CAT, catalase; APX, ascorbate peroxidase; PPO, polyphenol oxidase; LOX, lipoxygenase; MDA, malondialdehyde; TSS, total soluble solids; TA, titratable acidity.

A third, clearly separated cluster included TPC, TFC, and ABTS, which were strongly and positively correlated (*r* = 0.80–0.90), confirming that antioxidant activity was closely associated with the accumulation of phenolic compounds during storage.

Finally, color coordinates clustered together and were positively associated with firmness and TA, while showing predominantly negative correlations with oxidative stress markers.

Overall, the correlation heatmap confirmed that postharvest strawberry quality is governed by interconnected physiological processes rather than isolated quality traits. The hierarchical clustering highlighted four major interesting groups of variables associated with color and texture, antioxidant defense, oxidative damage, and antioxidant metabolites, providing an integrated view of the mechanisms underlying fruit quality evolution during cold storage. Interestingly, the correlation structure closely reflected the multivariate patterns identified by ASCA, confirming that oxidative stress‐related variables and antioxidant enzymes evolved in a coordinated manner during storage, whereas phenolic compounds constituted a distinct metabolic response.

Despite the promising results obtained in the present study, and the extensive body of literature demonstrating the effectiveness of edible coatings in preserving strawberry quality, their commercial application in berry postharvest management remains relatively limited. This apparent discrepancy can be attributed to several practical and technological challenges. First, coating performance depends on achieving a delicate balance between moisture barrier properties and gas permeability. Excessively dense coatings may restrict oxygen diffusion, promote anaerobic respiration, and consequently induce ethanol accumulation, off‐flavor development, and undesirable sensory modifications.^72^ Furthermore, large‐scale industrial implementation remains challenging due to formulation‐dependent performance, the need for uniform coating deposition on irregular fruit surfaces, and the integration of coating application into commercial packing lines.^72^ Therefore, successful commercial exploitation requires not only effective preservation but also careful optimization of coating formulation, application procedures, and fruit physiology. In this context, the integrated physiological approach adopted in the present study may contribute to a deeper understanding of the mechanisms underlying coating performance, thereby supporting the rational design and optimization of future edible coating systems.

## CONCLUSION

The CMC–alginate bilayer edible coating effectively delayed strawberry senescence during refrigerated storage by modulating oxidative metabolism and preserving cellular integrity. The coordinated enhancement of antioxidant enzymatic activity together with the reduction of oxidative damage markers indicates that the coating acted by regulating the physiological response to postharvest stress rather than simply reducing water loss. The integration of conventional analytical determinations with multivariate statistical approaches provided a comprehensive understanding of the mechanisms underlying fruit quality evolution. ASCA identified storage time as the dominant source of multivariate variation, while treatment and its interaction with storage time significantly modified the physiological trajectory of the fruit. Hierarchical correlation analysis further revealed coordinated relationships among antioxidant defense, oxidative damage, and quality‐related traits, confirming the interconnected nature of these physiological processes.

Although the present study demonstrates the considerable potential of edible coatings for preserving strawberry quality, their commercial adoption remains limited. In this context, the integrated physiological framework proposed contributes to a deeper understanding of the mechanisms underlying coating performance and may support the rational optimization of future edible coating systems for commercial postharvest applications.

## CONFLICT OF INTEREST

The authors declare that they have no known competing financial interests or personal relationships that could have appeared to influence the work reported in this paper.

## AUTHOR CONTRIBUTIONS

**Ilenia Pellicano:** Writing – original draft, Methodology, Formal analysis, Data curation. **Riccardo Riggi:** Writing – original draft, Methodology, Formal analysis, Data curation. **Elvira Ferrara:** Investigation, Formal analysis, Writing – review and editing. **Danilo Cice:** Investigation, Formal analysis, Writing – review and editing. **Margherita Modesti:** Writing – original draft, Supervision, Data curation. **Milena Petriccione:** Supervision, Funding acquisition, Resources, Conceptualization, Methodology, Writing – review and editing. **Andrea Bellincontro:** Supervision, Funding acquisition, Conceptualization, Writing – review and editing.

## Data Availability

Data are available in the article.
